# Adaptive Kalman Filter-Based SLAM in LiDAR-Degenerated Environments

**DOI:** 10.3390/s26030861

**Published:** 2026-01-28

**Authors:** Ran Ma, Tao Zhou, Liang Chen

**Affiliations:** State Key Laboratory of Information Engineering in Surveying, Mapping and Remote Sensing, Wuhan University, Wuhan 430079, China; ranma16@foxmail.com (R.M.); zhoutaowhu@whu.edu.cn (T.Z.)

**Keywords:** SLAM, 2D LiDAR, particle filter, adaptive kalman filter

## Abstract

Owing to the low cost, small size, and convenience for installation, 2D LiDAR has been widely used in mobile robots for simultaneous positioning and mapping (SLAM). However, traditional 2D LiDAR SLAM methods have low robustness and accuracy in LiDAR-degenerated environments. To improve the robustness of the SLAM method in such environments, an innovative SLAM method is developed, which mainly includes two parts, i.e., the front-end positioning and the back-end optimization. Specifically, in the front-end part, the AKF (adaptive Kalman filter) method is applied to estimate the pose of the mobile robot, zero bias of acceleration and gyroscope, lever arm length, and the mounting angle. The adaptive factor of the AKF can dynamically adjust the variance of the process and measurement noises based on the residual. In the back-end part, a particle filter (PF) is employed to optimize the pose estimation and build the map, where the pose domain constraint from the output of the front-end is introduced in the PF to avoid mismatch and enhance positioning accuracy. To verify the performance of the method, a series of experiments is carried out in four typical environments. The experimental results show that the positioning precision has been improved by about 61.3–97.9%, 35.7–99.0%, and 43.8–93.0% compared to the Karto SLAM, Hector SLAM, and Cartographer, respectively.

## 1. Introduction

Simultaneous localization and mapping (SLAM) technology was first proposed by Smith in 1986 [1]. There is an extensive range of applications of SLAM technology, especially in robotics [2,3]. The SLAM methods mainly include visual-SLAM and LiDAR-SLAM. Conventional vision-based approaches often rely on RGB or depth imagery; they can be susceptible to varying lighting conditions, occlusions, and limited depth resolution. Recent advancements have explored the integration of three-dimensional data representation and deep learning to overcome these constraints. Notably, Zhang et al. [4] proposed a methodology to combine the 3D graph deep learning and laser point cloud for intelligent rehabilitation. By fusing laser point cloud data with 3D graph convolutional networks, this method achieves robust spatial feature extraction and segmentation accuracy in complex environments. However, as noted in comparative studies, while such visual-based methods are cost-effective and rich in texture information, they fundamentally depend on ambient lighting conditions and distinct environmental features. They are susceptible to challenges such as photometric calibration errors and rolling shutter effects, and can experience significant performance degradation or even tracking failure in low-light, low-texture, or highly dynamic scenes [5,6,7,8]. In contrast, LiDAR-based SLAM provides direct, high-precision geometric measurements of the environment, which are largely invariant to lighting changes and yield accurate metric maps, though they may lack semantic richness and face challenges in feature-sparse environments.

The LiDAR sensors are classified into 2D and 3D LiDARs. Two-dimensional LiDAR captures data on a signal plane, making it ideal for autonomous mobile robots (AMRs). The application benefits from 2D LiDAR’s lower cost, reduced complexity, and lower data volume, thus simplifying data processing and increasing response times for basic navigation. Three-dimensional LiDAR captures data in three dimensions, providing a comprehensive view essential for applications such as autonomous vehicles. Three-dimensional LiDAR generates millions of data points per second, offering highly detailed environmental mapping crucial for precise navigation and complex tasks. The huge data volume of 3D LiDAR means complex data processing and slower response times for navigation. Thus, compared with 3D LiDAR, 2D LiDAR has been widely used on the AMRs for position and navigation.

The main existing methods for LiDAR SLAM include LiDAR-only SLAM and multi-sensor fusion SLAM. LiDAR-only SLAM methods include direct matching methods and feature matching methods. Direct matching methods directly utilize LiDAR scan points for pose estimation, and direct matching methods include matching based on geometric models and maps. For the matching methods based on geometric models, iterative closest point (ICP) and its improvements are the most popular methods [9]. The improved aspects of the ICP’s improvements include subset sampling (e.g., voxel grid filtering [10], histogram sampling, etc.), distance metrics (e.g., point-to-line [11], etc.), and computational efficiency (e.g., Fast ICP [12], Anderson Acceleration ICP [13], etc.). Although the improvements in the ICP make it faster and more accurate than the original algorithm, these methods still have a high dependence on the initial value, which makes it easy to fall into local extrema. The methods based on matching maps match the LiDAR scan points with the grid map or the submaps. The grid map and submap are constructed based on LiDAR scan points. Popular methods based on matching maps have included the Gmapping method [14] and the Cartographer method [15] until now. The map offers richer structural information than sparse individual scans. Thus, compared with the methods based on matching LiDAR scan points, methods based on matching maps have a higher matching accuracy. However, there is still the issue of it being easy to fall into the local extrema if the features of the environment are similar or the initial value is not accurate enough. Feature-based methods utilize the extracted fundamental geometric properties, such as lines, corners, curves, etc., to match and reduce the time consumed. The LiDAR odometry and mapping (LOAM) algorithm [16] is a typical LiDAR SLAM framework that extracts 3D planar and edge points. The optimization metric of the LOAM is to minimize the distance between point-to-line and point-to-plane. However, the feature-based methods also have a high dependence on the initial value, and the feature extraction also influences the optimization results. The introduction of the multi-sensor fusion SLAM is to improve the accuracy and reliability of the initial value and reduce the probability of falling into local extrema. The mainstream multi-sensor fusion SLAM methods are LiDAR-inertial methods. The LiDAR-inertial fusion methods include inertial measurement unit (IMU) odometry and IMU-wheel odometry-aided methods. IMU-aided SLAM methods utilize the IMU measurements to correct the non-linear motion distortions of the LiDAR scans and use the IMU pre-integration results as the initialization of the scan matching to improve the robustness [17]. However, there are still some problems with IMU-aided SLAM methods in the mobile robots’ application. The mobile robots move at slow speeds with frequent starts and stops. Under such conditions, an accurate zero velocity update (ZUPT) is necessary for the IMU odometer, but it is difficult to judge the zero velocity status and estimate linear velocity accurately only based on a single IMU [18]. Thus, it still has low robustness when the IMU-aided SLAM methods work in LiDAR-degenerated environments, such as corridor environments. To solve this problem, the wheel odometer is introduced into the IMU-aided SLAM methods. For example, G.P.C. Júnior et al. [19] proposed the EKF-LOAM (extended Kalman filter-LiDAR odometry and mapping) method to solve the issues that the traditional LiDAR SLAM algorithms have difficulty mapping in the common industrial confined spaces, such as ducts and galleries, which have long and homogeneous structures. The experiments show that the EKF-LOAM method improves the positioning accuracy by more than 50% compared with the original LeGO-LOAM (lightWeight and ground optimized-LOAM) algorithm. However, this work utilized the 3D LiDAR in the proposed methods, and it did not explore the multi-sensor fusion methods based on 2D LiDAR. Thus, we proposed a method fusing IMU, wheel odometer, and 2D LiDAR to improve the robustness of 2D LiDAR SLAM in LiDAR-degenerated environments and address the challenge of high dependence on external environmental features for LiDAR SLAM methods. The main contributions of this work are as follows:

- A new adaptive Kalman filter (AKF) method is proposed to fuse IMU and the wheel odometer, which can estimate IMU’s acceleration and gyroscope zero biases, the mounting angle, and the lever arm length. The adaptive factor of the AKF can dynamically adjust the variance of the process noise and measurement noise based on the residual.
- In the back-end, the pose from AKF is introduced as constraints into the particle filter (PF) to overcome the mismatch, which commonly occurs in scan-map matching, especially under LiDAR-degenerated environments.
- The field tests show that the proposed method can provide a reliable and robust positioning and mapping service in LiDAR-degenerated environments, compared with the traditional 2D LiDAR SLAM methods (Karto SLAM, Hector SLAM, and Cartographer).

The organization of the paper is as follows: Section 2 mainly introduces the related works. Section 3 mainly introduces the system framework of the proposed method. Section 4 discusses the context of the adaptive Kalman filter, and Section 5 discusses the context of the back-end SLAM part. Section 6 introduces experimental results, and Section 7 discusses the results of the experiments. Section 8 concludes the paper and provides an outlook on future research directions.

## 2. Related Works

The prior works on LiDAR SLAM in LiDAR-degenerated environments are extensive. The works mainly include multi-sensor fused SLAM and utilizing artificial intelligence (AI) SLAM for point cloud registration or multi-sensor fusion. In this section, we briefly reviewed the work on these two aspects.

### 2.1. Multi-Sensors Fusion SLAM

The current LiDAR SLAM methods have proven to be accurate and robust enough in many environments, and here we focus on the performance of the LiDAR SLAM method in LiDAR-degenerated environments. Regarding these environments, researchers have found that adding auxiliary sensors or assisting geometric landmarks can improve the accuracy and robustness of LiDAR SLAM methods in LiDAR-degenerated environments.

Adding auxiliary sensors means introducing additional constraints. Cameras have proven to be good auxiliary sensors in some LiDAR degenerated environments or feature-sparse environments [20,21] but are still not accurate enough in LiDAR-degenerated environments, and the methods fusing cameras need more time than the traditional LiDAR SLAM methods. Ultra-wideband (UWB) technology is an environment-insensitive sensor, and the positioning error of the UWB does not accumulate with the increase in mileage. Thus, LiDAR SLAM fusing the UWB has attracted more and more interest in recent years [22]. However, for the LiDAR SLAM methods fusing the UWB, it is necessary to place the UWB stations in advance, and it needs large enough scenarios to build a good enough geometric configuration for the positioning methods based on UWB. LiDAR SLAM methods fusing inertial sensors are another mainstream method that can improve the accuracy and robustness of the LiDAR SLAM methods in LiDAR-degenerated environments. Qing et al. [23] utilized IMU to aid laser scan matching and used the positioning results of the IMU-aided scan matching methods to transform the laser scan, finally, matching the laser scan with the orthogonal weighted occupancy likelihood map to obtain the final positioning results. This method has a high accuracy in the library, but it does not discuss the performance of the proposed method in LiDAR-degenerated environments, such as corridors. Chen et al. [24] utilized factor graph optimization to fuse the IMU and wheel odometer and used the positioning results to transform the laser scan to map coordinate systems. Reference [24] also utilized the visual landmarks to improve the accuracy. This method has high accuracy in LiDAR-degenerated environments but is highly dependent on visual landmarks.

Beyond these specific implementations, the factor graph optimization framework has become the standard method for achieving tightly-coupled, high-precision multi-sensor fusion in modern SLAM systems [25,26]. This framework elegantly unifies heterogeneous measurements—including IMU pre-integration, LiDAR odometry, loop closures, and absolute measurements from sources like GPS or landmarks as probabilistic constraints within a unified graph. Representative works, such as LIO-SAM [25] and reference [26], demonstrate the strength of this approach by deeply integrating LiDAR features with IMU data in a tightly-coupled manner, achieving state-of-the-art accuracy and robustness. The primary advantage of factor graph-based fusion lies in its flexibility and ability to perform global batch or sliding-window optimization, which optimally balances information from all sensors over time. However, this comes at the cost of growing computational complexity with the trajectory length, posing challenges for resource-constrained platforms or lifelong operation, where real-time performance is critical.

### 2.2. AI-Based SLAM

With the development of AI, using artificial intelligence models or data-driven approaches to solve the LiDAR-based SLAM problem has become a new trend. For AI-based SLAM, C. Li et al. [27] utilized the recurrent convolutional neural network (RCNN) to fuse IMU and 2D LiDAR and used an ICP-based scan-to-submap method to optimize the pose. This method has a higher accuracy compared with the traditional Hector SLAM. Nicolai et al. [28] utilized convolution neural networks (CNNs) to reduce the state space of laser scans and address the challenge of real-time positioning in large environments. Deng et al. [29] introduced the point pair feature network (PPNET), which leverages deep learning to process global 3D point clouds to solve the issue of finding correspondences in unorganized point clouds. Vongkubihisal et al. [30] presented the inverse composition discriminative optimization for 3D point cloud matching to solve the problem that traditional local point cloud registration methods are often affected by the presence of noise, outliers, and poor initialization. These all prove that AI technology has injected new vitality into the SLAM methods, but a large enough dataset is needed to train the model. Moreover, until now, AI-based methods still cannot reach the accuracy and reliability of traditional methods in some conditions.

## 3. System Overview

**Figure 1:**
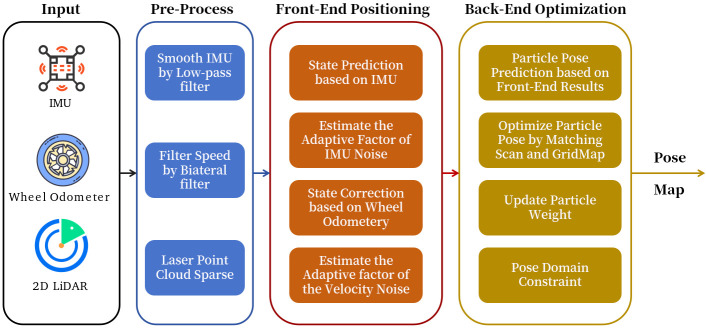
Overview of proposed method.

The proposed system includes data pre-processing, front-end positioning, and back-end optimization. The proposed method’s input includes an IMU, wheel odometer, and 2D LiDAR, and its output is the mobile robot’s pose and a global map. Figure 1 shows the system overview. The data pre-process step filters the raw sensor data, which includes three parts: (1) filtering the wheel speed based on the bilateral filter [31]; (2) filtering the IMU based on the low-pass filter [32,33]; (3) downsampling the laser point cloud using the voxel filter [34].

Regarding the front-end positioning, we utilized AKF to fuse the IMU and wheel odometer and estimate the mobile robot’s pose. The positioning results from the front-end are not accurate enough to enable map construction. To improve the positioning accuracy and build the map, we utilized the scan-map matching method to estimate the accurate yaw angle and position and used the particle filter to fuse these results with the front-end positioning data. To improve the system’s robustness in complex environments, we introduced the pose domain constraint to the back-end SLAM.

## 4. Adaptive Kalman Filter

### 4.1. System Model

The system model in this work can be expressed as

(1) $$x_{k}=f\left(x_{k-1}\right)+w_{k}$$

(2) $$z_{k}=h\left(x_{k}\right)+v_{k}$$

where $x_{k}\in R^{n}$ is the state vector, and $z_{k}\in R^{m}$ is the measurement vector, including the linear velocity observation from the wheel odometer. f(·) is the transforming function, and h(·) is the observation function. $w_{k}$ and $v_{k}$ are the process and measurement noise, respectively, and they are assumed to be zero-mean Gaussian white with covariance matrix $Q_{k}$, $P_{k}$, i.e., $w_{k}∼N\left(0,Q_{k}\right)$ and $v_{k}∼N\left(0,R_{k}\right)$.

The state vector $x_{k}$ is defined as

(3) $$x_{k}={\left(p_{3\times 1}^{n},v_{3\times 1}^{n},\theta_{b3\times 1}^{n},b_{a3\times 1},b_{g3\times 1},\theta_{i3\times 1}^{b},r_{3\times 1}\right)}^{T}$$

where *n* represents the navigation coordinate system, and *b* denotes the body coordinate system. $p_{3\times 1}^{n}$ is the three-axis position in the *n* system, and $v_{3\times 1}^{n}$ is three-axis velocity in the *n* system. $\theta_{b3\times 1}^{n}$ represents the attitude angle. $b_{g3\times 1}$ represents zero bias of the IMU gyroscope. $b_{a3\times 1}$ is zero bias of IMU acceleration. $r_{3\times 1}$ here represents the IMU mounting lever arm, and $\theta_{i3\times 1}^{b}$ is the IMU mounting angle in the body coordinate system. Based on the IMU preintegration, f(·) is expressed as follows:

(4) $$p_{k}=p_{k-1}+v_{k-1}\Delta t$$

(5) $$v_{k}=v_{k-1}+f_{k}\Delta t$$

(6) $$f_{k}=C_{b,k-1}^{n}\left(C_{i,k-1}^{b}f_{i,k}-b_{a,k-1}-Mr_{k-1}\right)-g$$

(7) $$\theta_{b,k}^{n}=\theta_{b,k-1}^{n}+\left(\omega_{k}-b_{g,k-1}\right)\Delta t$$

(8) $$b_{g,k}=b_{g,k-1}$$

(9) $$b_{a,k}=b_{a,k-1}$$

(10) $$\theta_{i,k}^{b}=\theta_{i,k-1}^{b}$$

(11) $$r_{k}=r_{k-1}$$

where $p_{k}$ is the position at the *k*th epoch, and $p_{k-1}$ is the position at the k−1th epoch. $v_{k-1}$ denotes the velocity at the k−1th epoch, and $v_{k}$ denotes the velocity at the *k*th epoch. $C_{b}^{n}$ and $C_{i}^{b}$ are rotation matrices representing the transformation from the body coordinate system to the navigation coordinate system and from the IMU coordinate system to the body coordinate system, respectively. $C_{i}^{b}$ can be transformed from the Euler angle $\theta_{i}^{b}$, and $C_{b}^{n}$ can be transformed according the Euler angle $\theta_{b}^{n}$. $f_{i,k}$ denotes the acceleration measurements at the *k*th epoch, and $b_{a,k-1}$ denotes the zero bias of the acceleration at the k−1th epoch. *M* represents the scale matrix for tangential and centripetal acceleration due to rotation, which can be estimated by the measurement of the IMU gyroscope, and the estimation process can be expressed as (12). $r_{k-1}$ represents the mounting arm of the IMU at the k−1th epoch, and g represents the earth gravity vector. $\theta_{b,k}^{n}$ is the attitude angle at the *k*th epoch, and $\theta_{b,k-1}^{n}$ is the attitude angle at the k−1th epoch. $\omega_{k}$ denotes the gyroscope measurements at the *k*th epoch, and $b_{g,k-1}$ denotes the zero bias of the gyroscope at the k−1th epoch.

(12) $$M={\dot{\omega }}_{ib}\times +\omega_{ib}\times \left(\omega_{ib}\times \right)=\left(\begin{array}{ccc} -(\omega_{y}^{2}+\omega_{z}^{2}) & \omega_{x}\omega_{y}-{\dot{\omega }}_{z} & \omega_{x}\omega_{z}+{\dot{\omega }}_{y}\\ \omega_{x}\omega_{y}+{\dot{\omega }}_{z} & -(\omega_{x}^{2}+\omega_{z}^{2}) & \omega_{y}\omega_{z}-{\dot{\omega }}_{x}\\ \omega_{x}\omega_{z}-{\dot{\omega }}_{y} & \omega_{y}\omega_{z}+{\dot{\omega }}_{x} & -(\omega_{x}^{2}+\omega_{y}^{2}) \end{array}\right)$$

where ${\dot{\omega }}_{ib}\times$ is the scale factor of tangential acceleration due to rotation motion, and $\omega_{ib}\times$ denotes the scale factor of centripetal acceleration due to rotation motion. ${\left({\dot{\omega }}_{x},{\dot{\omega }}_{y},{\dot{\omega }}_{z}\right)}^{T}$ represents the time derivatives of the angular velocities. The measurement vector $z_{k}$ can be modeled as

(13) $$z_{k}=C_{b,k-1}^{n}v_{k}$$

where $v_{k}$ is the linear velocity measurement provided by the wheel odometer at the *k*th epoch. $C_{b,k-1}^{n}$ denotes the quaternion transforming from the body frame to the navigation frame at the *k*th epoch. Thus, the observation function h(·) can be expressed as matrix *H*, which is as follows:

(14) $$H=\left(\begin{array}{ccccccc} 0_{3\times 3} & I_{3\times 3} & 0_{3\times 3} & 0_{3\times 3} & 0_{3\times 3} & 0_{3\times 3} & 0_{3\times 3} \end{array}\right)$$

To sum up, the problem of estimating the position and velocity of the mobile robot is to infer the current mobile state $x_{k}$ from the observation sequence $z_{1:k}$. Within the framework of Bayesian inference, the problem corresponds to computing the marginal posterior $p(x_{k}|z_{1:k})$. It has been demonstrated that the Kalman filter (KF) can achieve optimal estimation in a linear state model with Gaussian white noise. However, in (5), the transforming process of the state components of the gyroscope zero bias and the IMU mounting angle is nonlinear, which limits the application of KF. Hence, in our state estimation problem, we resorted to the EKF method, which can effectively solve the non-linear optimization problem [35]. In addition, the uncertainty for the covariance of the processing noise and measurement noise will influence the robustness and accuracy of the filter, so the adaptive factors are introduced into the EKF model to obtain the adaptive Kalman filter (AKF). In this section, the AKF will be detailed.

### 4.2. State Estimation with AKF

The AKF includes two steps, and it can be summarized as follows:

#### 4.2.1. State Prediction

Based on the state model, the state prediction can be calculated as

(15) $${\hat{x}}_{k|k-1}=\Phi_{k|k-1}{\hat{x}}_{k-1}+{\hat{c}}_{x,k-1}$$

(16) $${\hat{P}}_{k|k-1}=\Phi_{k|k-1}{\hat{P}}_{k-1}\Phi_{k|k-1}^{T}+G_{k-1}Q_{k-1}G_{k-1}^{T}$$

where ${\hat{x}}_{k|k-1}$ represents the prior state estimation at the *k*th epoch, and ${\hat{x}}_{k-1}$ represents posterior state estimation at the k−1th epoch. $\Phi_{k|k-1}$ denotes the state transformer matrix at the *k*th epoch, and ${\hat{c}}_{x,k-1}$ denotes the adaptive factor that is utilized to adjust the processing noise covariance matrix $Q_{k-1}$. ${\hat{P}}_{k|k-1}$ is the prior state error covariance matrix at the *k*th epoch, and ${\hat{P}}_{k-1}$ is the posterior state error covariance matrix at the k−1th epoch. $G_{k-1}$ represents the processing error transform matrix at the k−1th epoch. The adaptive factor ${\hat{c}}_{x,k-1}$ is expressed as

(17) $${\hat{c}}_{x,k-1}=\frac{1}{L}\sum_{l=k-L}^{k-1}\beta_{l}\left({\hat{x}}_{l}-{\hat{x}}_{l|l-1}\right)$$

(18) $$\beta_{l}=\frac{b^{L-l}\left(1-b\right)}{1-b^{L}},l=1,2,\ldots ,L$$

where *L* is the length of the sliding window, and $\beta_{l}$ is the weight of the adaptive element $\left({\hat{x}}_{l}-{\hat{x}}_{l|l-1}\right)$. *b* denotes the Suge forgetting factor, and *b* is satisfied with 0<b<1. The state transform matrix $\Phi_{k|k-1}$, processing noise covariance matrix $Q_{k-1}$, and the processing error transform matrix $G_{k-1}$ are expressed as

(19) $$\Phi_{k|k-1}=\left(\begin{array}{ccccccc} I_{3\times 3} & I_{3\times 3}\Delta t & \; & & \; & & \\ \; & I_{3\times 3} & \; & \frac{\partial (f_{k}+{\dot{f}}_{k}\Delta t)}{\partial b_{a}}\Delta t & \; & \frac{\partial (f_{k}+{\dot{f}}_{k}\Delta t)}{\partial \theta_{i}^{b}}\Delta t & \frac{\partial (f_{k}+{\dot{f}}_{k}\Delta t)}{\partial r}\Delta t\\ \; & \; & I_{3\times 3} & \; & -I_{3\times 3}\Delta t & \; & \\ \; & \; & & I_{3\times 3} & \; & & \\ \; & \; & & I_{3\times 3} & \; & & \\ \; & \; & & \; & I_{3\times 3} & \; & \\ \; & \; & & \; & & I_{3\times 3} & \\ \; & \; & & \; & & \; & I_{3\times 3} \end{array}\right)$$

(20) $$Q_{k-1}=diag\left(\sigma_{f}^{2}I_{3\times 3},\sigma_{\omega }^{2}I_{3\times 3},\sigma_{ba}^{2}I_{3\times 3},\sigma_{bg}^{2}I_{3\times 3},\sigma_{\theta_{i}^{b}}^{2}I_{3\times 3},\sigma_{r}^{2}I_{3\times 3}\right)$$

(21) $$G=\left(\begin{array}{cccccc} \frac{1}{2}C_{b,k-1}^{n}C_{i,k-1}^{b}\Delta t^{2} & \; & & \; & & \\ \frac{\partial (f_{k}+{\dot{f}}_{k}\Delta t)}{\partial f_{i}}\Delta t & \; & \frac{\partial (f_{k}+{\dot{f}}_{k}\Delta t)}{\partial b_{a}}\Delta t & \; & \frac{\partial (f_{k}+{\dot{f}}_{k}\Delta t)}{\partial \theta_{i}^{b}}\Delta t & \frac{\partial (f_{k}+{\dot{f}}_{k}\Delta t)}{\partial r}\Delta t\\ \; & I_{3\times 3}\Delta t & \; & -I_{3\times 3}\Delta t & \; & \\ \; & \; & I_{3\times 3} & \; & & \\ \; & \; & & I_{3\times 3} & \; & \\ \; & \; & & \; & I_{3\times 3} & \\ \; & \; & & \; & & I_{3\times 3} \end{array}\right)$$

(22) $$\frac{\partial (f_{k}+{\dot{f}}_{k}\Delta t)}{\partial f_{i}}\Delta t=\left(C_{b,k-1}^{n}\Delta t+\omega_{k}^{\wedge }C_{b,k-1}^{n}\Delta t^{2}\right)C_{i,k-1}^{b}$$

(23) $$\frac{\partial (f_{k}+{\dot{f}}_{k}\Delta t)}{\partial b_{a}}\Delta t=-C_{b,k-1}^{n}\Delta t-\omega_{k}^{\wedge }C_{b,k-1}^{n}\Delta t^{2}$$

(24) $$\frac{\partial (f_{k}+{\dot{f}}_{k}\Delta t)}{\partial \theta_{i}^{b}}\Delta t=C_{b,k-1}^{n}\frac{\partial (C_{i,k-1}^{b}f_{i,k})}{\partial \theta_{i,k}^{b}}\Delta t+\omega_{k}^{\wedge }C_{b,k-1}^{n}\frac{\partial (C_{i,k-1}^{b}f_{i,k})}{\partial \theta_{i,k}^{b}}\Delta t^{2}$$

(25) $$\frac{\partial (f_{k}+{\dot{f}}_{k}\Delta t)}{\partial r}\Delta t=-C_{b,k-1}^{n}M\Delta t-\omega_{k}^{\wedge }C_{b,k-1}^{n}M\Delta t^{2}$$

(26) $$\omega_{k}^{\wedge }=\left(\begin{array}{ccc} 0 & -\omega_{z} & \omega_{y}\\ \omega_{z} & 0 & -\omega_{x}\\ -\omega_{y} & \omega_{x} & 0 \end{array}\right)$$

where $\sigma_{f}^{2}$ denotes the error sigma of the acceleration measurements, $\sigma_{\omega }^{2}$ denotes the error sigma of the gyroscope measurements. $\sigma_{ba}^{2}$ represents the error sigma of the zero bias of the acceleration, and $\sigma_{bg}^{2}$ represents the error sigma of the zero bias of the gyroscope. $\sigma_{\theta_{i}^{b}}^{2}$ is the error sigma of the IMU mounting angle, and $\sigma_{r}^{2}$ is the error sigma of the IMU mounting lever arm. *M* represents the scale matrix for tangential and centripetal acceleration due to rotation, which is expressed as (12). $f_{i,k}$ denotes the acceleration measurement, and $\omega_{k}={\left(\omega_{x},\omega_{y},\omega_{z}\right)}_{k}^{T}$ denotes the gyroscope measurement.

#### 4.2.2. State Update

The state updated is calculated as follows:

(27) $$K_{k}=\left({\hat{P}}_{k|k-1}H^{T}\right){\left(H{\hat{P}}_{k|k-1}H^{T}+R\right)}^{-1}$$

(28) $${\hat{x}}_{k}={\hat{x}}_{k|k-1}+K_{k}\left(z_{k}-H{\hat{x}}_{k|k-1}-{\hat{c}}_{z,k-1}\right)$$

(29) $${\hat{P}}_{k}={\hat{P}}_{k|k-1}-K_{k}H{\hat{P}}_{k|k-1}$$

where $K_{k}$ represents the Kalman filter gain, and *H* represents the measurement transform matrix, which is expressed as $H=(0_{3\times 3},I_{3\times 3},0_{3\times 3},0_{3\times 3},0_{3\times 3},0_{3\times 3},0_{3\times 3})$. ${\hat{c}}_{z,k-1}$ denotes the adaptive factor that is utilized to adjust the error covariance matrix of the measurements *R*, and ${\hat{c}}_{z,k-1}$ is expressed as

(30) $${\hat{c}}_{z,k-1}=\frac{1}{L}\sum_{l=k-L}^{k-1}\beta_{l}\left(z_{l}-H{\hat{x}}_{l|l-1}\right)$$

where *L* represents the length of the sliding window, and $\beta_{l}$ represents the weight of the adaptive element $\left(z_{l}-H{\hat{x}}_{l|l-1}\right)$, and it is expressed as $\beta_{l}=\frac{b^{L-l}\left(1-b\right)}{1-b^{L}},l=1,2,\ldots ,L$.

## 5. PF in the Back-End Optimization

In the back-end optimization, we further improved the positioning accuracy from the front-end and built the grid probability map based on PF. The particle state is modeled as

(31) $$x_{k,PF}^{\left(j\right)}={\left(p_{x,k}^{\left(j\right)},p_{y,k}^{\left(j\right)},\theta_{k}^{\left(j\right)}\right)}^{T}$$

where $x_{k,PF}^{\left(j\right)}$ is the particle *j* state at *k*th epoch. ${\left(p_{x,k}^{\left(j\right)},p_{y,k}^{\left(j\right)}\right)}^{T}$ is the 2D position of the particle *j* at *k*th epoch. $\theta_{k}^{\left(j\right)}$ is the yaw of the particle *j* at the *k*th epoch. The particle sampling is modeled as

(32) $$x_{k|k-1,PF}^{\left(j\right)}=x_{k-1,PF}^{\left(j\right)}+u_{k|k-1}^{\left(j\right)}+n_{k-1}^{\left(j\right)}$$

where $x_{k|k-1,PF}^{\left(j\right)}$ denotes the predicted particle *j* state at the *k*th epoch, and $x_{k-1,PF}^{\left(j\right)}$ denotes the particle *j* state at the k−1th epoch. $u_{k|k-1}^{\left(j\right)}$ represents the odometer measurement from the front-end, and $n_{k-1}^{\left(j\right)}$ represents the odometer measurement noise, which is generated as a zero-mean Gaussian distribution. $u_{k|k-1}^{\left(j\right)}$ is expressed as

(33) $$u_{k|k-1}^{\left(j\right)}=\left(\begin{array}{c} d_{k|k-1}\cos\theta_{k-1}^{\left(j\right)}\\ d_{k|k-1}\sin\theta_{k-1}^{\left(j\right)}\\ \theta_{k|k-1} \end{array}\right)$$

where $d_{k|k-1}$ represents the move distance from the front-end, and it can be expressed as (34). $\theta_{k|k-1}$ denotes the yaw change from the front-end, and it can be expressed as (35).

(34) $$d_{k|k-1}=\sqrt{{\left({\hat{p}}_{x,k}-{\hat{p}}_{x,k-1}\right)}^{2}+{\left({\hat{p}}_{y,k}-{\hat{p}}_{y,k-1}\right)}^{2}}$$

where ${\left({\hat{p}}_{x,k},{\hat{p}}_{y,k}\right)}^{T}$ is the mobile position in *m* system at the *k*th epoch, and ${\left({\hat{p}}_{x,k-1},{\hat{p}}_{y,k-1}\right)}^{T}$ is the mobile position in *m* system at the k−1th epoch.

(35) $$\theta_{k|k-1}={\hat{\theta }}_{b,k}^{n}-{\hat{\theta }}_{b,k-1}^{n}$$

where ${\hat{\theta }}_{b,k}^{n}$ represents the robot’s yaw at the *k*th epoch, and ${\hat{\theta }}_{b,k-1}^{n}$ represents the robot’s yaw at k−1th epoch. ${\hat{\theta }}_{b,k}^{n}$ and ${\hat{\theta }}_{b,k-1}^{n}$ are from $q_{b,k}^{n}$ and $q_{b,k-1}^{n}$, separately. Normally, to improve the accuracy of the particle pose, the predicted particle pose $x_{k|k-1,PF}^{\left(j\right)}$ is to be optimized by a scan-map matcher, and the optimization is based on the ICP method [9,36]. The optimized particle pose is denoted as $x_{k|k-1,PF}^{(j)-}$. The particle weight is calculated as

(36) $${\tilde{w}}^{\left(j\right)}=\exp\left\{-\sum_{i}\frac{{\left(l_{x,i}^{\left(j\right)}-{\bar{l}}_{x,i}\right)}^{2}+{\left(l_{y,i}^{\left(j\right)}-{\bar{l}}_{y,i}\right)}^{2}}{\sigma^{2}}\right\}$$

where σ represents the standard deviation of the LiDAR data, which is normally equal to the resolution of the grid map. ${\left({\bar{l}}_{x,i},{\bar{l}}_{y,i}\right)}^{T}$ denotes the mean coordinate of the historical LiDAR points in the *i*th grid. ${\left(l_{x,i}^{\left(j\right)},l_{y,i}^{\left(j\right)}\right)}^{T}$ denotes the LiDAR point at the current frame, which falls within the *i*th grid. ${\left(l_{x,i}^{\left(j\right)},l_{y,i}^{\left(j\right)}\right)}^{T}$ is obtained by transforming the original laser point from *b* system to *m* system based on the particle *j* state $x_{k|k-1,PF}^{(j)-}$, and the transforming process can be expressed as

(37) $$\left(\begin{array}{c} l_{x}^{\left(j\right)}\\ l_{y}^{\left(j\right)} \end{array}\right)=\left(\begin{array}{ccc} \cos\theta^{(j)-} & -\sin\theta^{(j)-} & p_{x}^{(j)-}\\ \sin\theta^{(j)-} & \cos\theta^{(j)-} & p_{y}^{(j)-} \end{array}\right)\left(\begin{array}{c} l_{x}\\ l_{y}\\ 1 \end{array}\right)$$

where ${\left(l_{x},l_{y}\right)}^{T}$ is the original laser point, and ${\left(l_{x}^{\left(j\right)},l_{y}^{\left(j\right)}\right)}^{T}$ is the transformed laser point. The particle quantity is calculated as

(38) $$N_{eff}=\frac{1}{\sum_{j=1}^{J}{\left({\bar{\tilde{w}}}^{\left(j\right)}\right)}^{2}}$$

where *J* is the particles’ number, and ${\bar{\tilde{w}}}^{\left(j\right)}$ is the normalized weight of the particle *j*. We resampled the particle when the $N_{eff}$ drops below the threshold J/2. The optimized results of the PF are as

(39) $$x_{k,PF}=\sum_{j=1}^{J}{\bar{\tilde{w}}}^{\left(j\right)}x_{k|k-1,PF}^{(j)-}$$

To improve the robustness, the pose from AKF is introduced as the constraint, and the process is as

(40) $$\left(\begin{array}{c} p_{x,k|k-1}^{(j)-}\\ p_{y,k|k-1}^{(j)-} \end{array}\right)=\left(\begin{array}{c} p_{x,k-1}^{\left(j\right)}\\ p_{y,k-1}^{\left(j\right)} \end{array}\right)+\left(\begin{array}{c} d_{k|k-1}\cos\theta_{k-1}^{\left(j\right)}\\ d_{k|k-1}\sin\theta_{k-1}^{\left(j\right)} \end{array}\right),\left|d_{k|k-1}-d_{k|k-1,PF}\right|>t_{d}$$

(41) $$\theta_{k|k-1}^{(j)-}=\theta_{k-1}^{\left(j\right)}+\theta_{k|k-1},\left|\theta_{k|k-1}-\theta_{k|k-1,PF}\right|>t_{\theta }$$

where $d_{k|k-1,PF}$ denotes the move distance from PF, and $\theta_{k|k-1,PF}$ denotes the yaw change from PF. $t_{d}$ and $t_{\theta }$ are thresholds. After (40) and (41), the PF results $x_{k,PF}$ will be updated according to (39), and the grid map will be extended based on $x_{k,PF}$.

## 6. Experimental Results

### 6.1. Experiments Settings

#### 6.1.1. Experiment Platform

**Figure 2:**
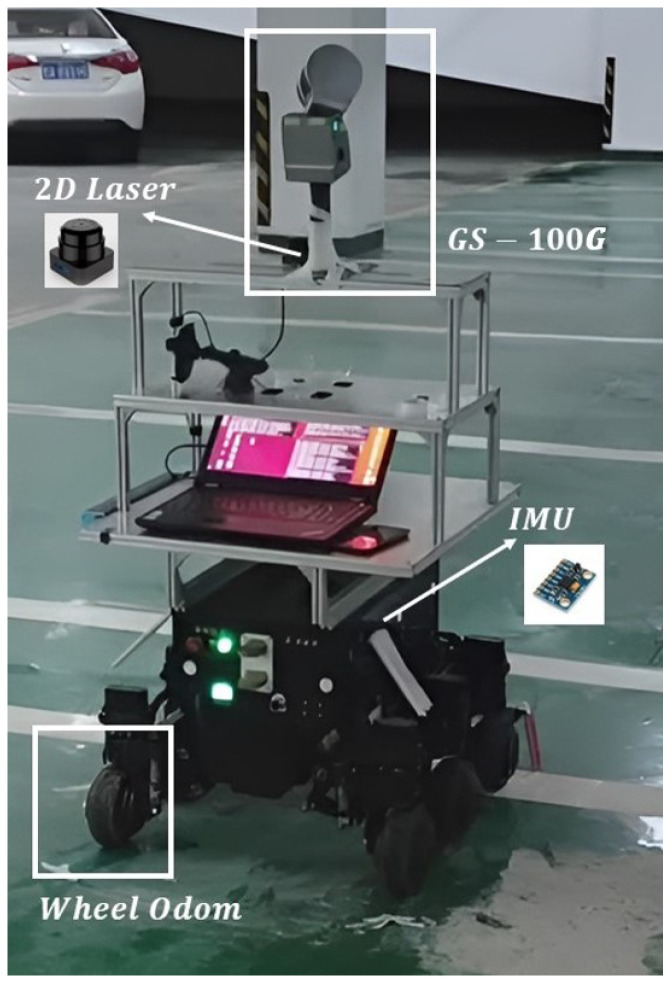
Experimental platform.

To evaluate the proposed method’s positioning performance, a six-wheel robot has been utilized for data recording, which is equipped with an IMU, wheel odometer, and 2D LiDAR. The sensor configurations are shown in Table 1. A GS-100G laser scanner, which is from the GEOSUN company in China, provides the reference trajectory. According to [37], the horizontal position accuracy is 0.02 m, and the elevation position accuracy is 0.03 m, which illustrates that the accuracy of the reference trajectory is satisfactory with the experimental evaluation. The overall experiment platform is shown in Figure 2.

#### 6.1.2. Experiment Scenarios

**Figure 3:**
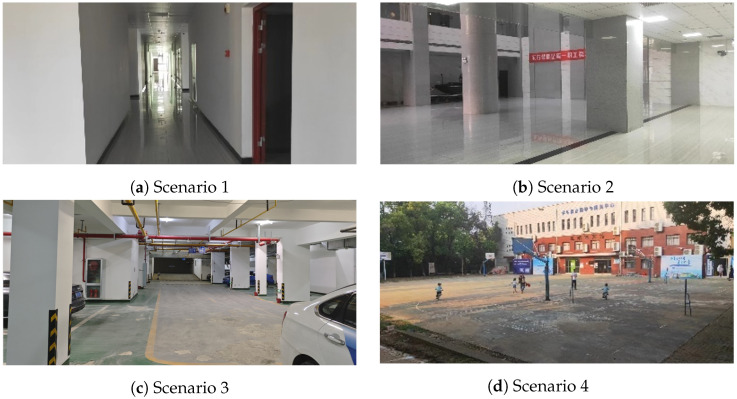
Experimental Scenarios. Scenario 1 is a corridor, Scenario 2 is a hall, Scenario 3 is a parking lot, and Scenario 4 is a playground.

The experiments were carried out in four scenarios, which include a corridor, hall, park, and playground, and the experiment scenarios are shown in Figure 3. As shown in Figure 3a, the corridor is approximately 3 m wide and 50 m long, and the valid measurement range of the 2D LiDAR is less than the length of the corridor, which might be a LiDAR degenerated environment. As shown in Figure 3b, the hall is approximately 14 m wide and 18 m long, and there are rich geometric features and a good closure. As shown in Figure 3c, the parking lot belongs to a semi-open environment. The playground is approximately 32 m wide and 50 m long, and the scenario is relatively open, with sparse geometric objects, as shown in Figure 3d. The selected playground also belongs to the LiDAR-degenerated environment. Recording for the quantitative standards for LiDAR degradation environments, this section is based on the following [38]:

(42) $$x_{i}^{\prime }=x_{i}-\frac{1}{\lambda N}\sum_{j=1}^{N}x_{j}$$

(43) $$y_{i}^{\prime }=y_{i}-\frac{1}{\lambda N}\sum_{j=1}^{N}y_{j}$$

(44) $$k=\frac{\sum_{i}^{N}x_{i}^{\prime }}{\sum_{i}^{N}y_{i}^{\prime }}$$

(45) $$\mu =-\frac{1}{k}+1$$

where ${\left(x_{j},y_{j}\right)}^{T}$ represents the coordinate of each point in the current laser scan, and *N* represents the number of points in the current laser scan. ${\left(x_{i}^{\prime },y_{j}^{\prime }\right)}^{T}$ is the coordinate of each point in the centroid coordinates of the entire point cloud. In actuality, the range of *k* is [1,∞], and it is mapped to the interval [0,1] to obtain the degeneration degree μ. λ is the scale value, which is set as 5 in this work. When the value of μ is close to 1, it demonstrates a higher degree of degeneration. The mean values of μ in the selected scenarios are counted in Table 2.

To make the degree of degeneration more obvious, the values of Table 2 are normalized to range [0,1] among Scenarios 1–4. According to Table 2, it is found that Scenario 1 and Scenario 4 have a higher degree of degeneration compared with that of Scenario 2 and Scenario 3.

#### 6.1.3. Evaluation Metrics

The evaluation metrics include the maximum, mean, and median errors, as well as the root mean square error (RMSE), which is calculated as

(46) $$RMSE=\sqrt{\frac{1}{K}\sum_{k=1}^{K}{\left(p_{e,k}-p_{r,k}\right)}^{T}\left(p_{e,k}-p_{r,k}\right)}$$

where $p_{e,k}$ represents the estimated position. $p_{r,k}$ is the reference position. *K* denotes the number of epochs.

#### 6.1.4. System Parameter Settings

In this work, the *Q* is modeled as

(47) $$Q=\mathrm{diag}{\left(I_{3\times 3}\cdot \sigma_{bg}^{2},I_{3\times 3}\cdot \sigma_{ba}^{2},I_{3\times 3}\cdot \sigma_{l}^{2},I_{3\times 3}\cdot \sigma_{\theta }^{2}\right)}^{T}$$

where $\sigma_{bg}^{2}$ represents the noise sigma of the zero bias of the gyroscope, and $\sigma_{ba}^{2}$ represents the noise sigma of the zero bias of the accelerometer. $\sigma_{l}^{2}$ denotes the noise sigma of the IMU mounting lever arm, and $\sigma_{\theta }^{2}$ denotes the noise sigma of the IMU mounting angle. *R* is modeled as

(48) $$R=I_{3\times 3}\cdot \sigma_{v}^{2}$$

where $\sigma_{v}$ refers to the noise sigma of the linear velocity measurement from the wheel odometer.

In this work, the parameter settings are as follows: ${\hat{\sigma }}_{bg}=4.8\times 10^{-5}\;\mathrm{rad}/s$, ${\hat{\sigma }}_{ba}=0.343\;m/s^{2}$, ${\hat{\sigma }}_{l}=0.01\;m$, ${\hat{\sigma }}_{\theta }=1^{{}^{\circ}}$, and ${\hat{\sigma }}_{v}=0.0017\;m/s$.

About the setting of the noise sigma of the accelerometer (${\hat{\sigma }}_{ba}$) and the gyroscope’s zero biases (${\hat{\sigma }}_{bg}$), they are set according to the zero bias stability. According to Table 1, ${\hat{\sigma }}_{ba}$ is set as 0.343 $m/s^{2}$ (3500 μg), and ${\hat{\sigma }}_{bg}$ is set as $4.8\times 10^{-5}$ rad/s ($10^{{}^{\circ}}/$h). In this work, the IMU mounting angle and IMU mounting lever arm are obtained from the external parameter measurements. The accuracy of the distance measurement is about 0.01 m, and the angle measurement’s accuracy is about $1^{{}^{\circ}}$. Thus, ${\hat{\sigma }}_{l}$ is set as $1^{{}^{\circ}}$, ${\hat{\sigma }}_{\theta }$ is set as 0.01 m. $\sigma_{v}$ is modeled as

(49) $$\sigma_{v}=\frac{2\pi }{P\Delta t_{v}}\sigma_{r}$$

where *P* is the number of pulses rotated in a circle, $\Delta t_{v}$ is the time interval of the sampling. $\sigma_{r}$ denotes the measurement error of the wheel’s diameter. In this work, *P* is 360, $\Delta t_{v}$ is 0.01 s, and $\sigma_{r}$ is set as 0.01 m. Thus, $\sigma_{v}$ is set as about 0.0017 m/s. Regarding the PF parts, the particle counts are set to 30, the threshold $t_{d}$ is set to 1 m, and $t_{\theta }$ is set to 30°.

### 6.2. Positioning and Mapping Results

The algorithm comparison is shown in Table 3. The compared algorithms include Karto SLAM, Hector SLAM, and Cartographer. The Karto SLAM, Hector SLAM, and the proposed method utilized the AKF as the front-end, and the Cartographer utilized pre-integration as the front-end.

In this section, Karto SLAM is selected with the melodic-devel version, Hector SLAM is selected with Version 0.4.1, and Cartographer is selected with Cartographer_ros master. These algorithms are running on the computer with an Intel Core i7-13700KF, and random access memory with 32 GB. The environment of the computer is Ubuntu-18.04, and the version of ROS is Melodic.

#### 6.2.1. Mapping Results

**Figure 4:**
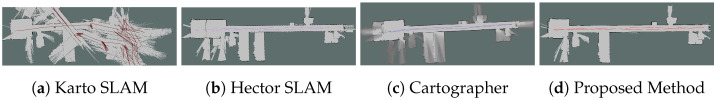
Mapping results in Scenario 1. The line in the figure represents the trajectory of the corresponding method.

**Figure 5:**
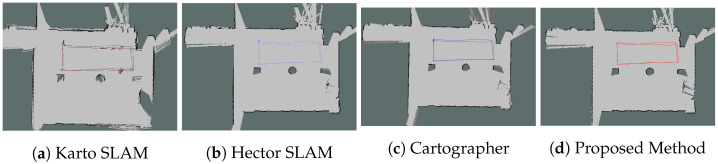
Mapping results in Scenario 2. The line in the figure represents the trajectory of the corresponding method.

**Figure 6:**
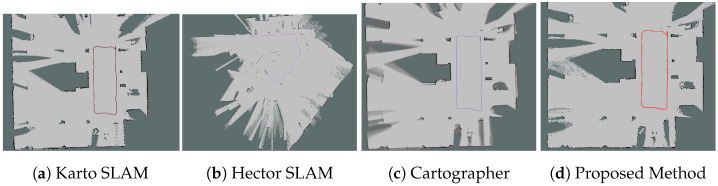
Mapping results in Scenario 3. The line in the figure represents the trajectory of the corresponding method.

**Figure 7:**
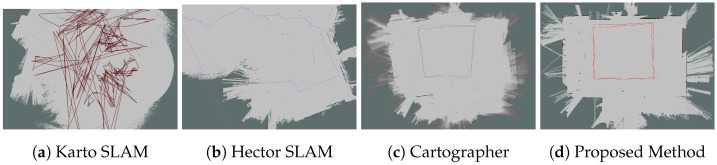
Mapping results in Scenario 4. The line in the figure represents the trajectory of the corresponding method.

From Figure 4, Figure 5, Figure 6 and Figure 7, we observed that the map from the proposed method has the highest accuracy compared with Karto SLAM, Hector SLAM, and Cartographer. In Scenario 1 and Scenario 4, it is observed that Karto SLAM, Hector SLAM, and Cartographer failed to build the map and localization, but the proposed method has built a relatively accurate map and provided a reliable positioning service, which suggests that the proposed method can provide a reliable mapping and positioning service in the LiDAR-degenerated environments. Figure 5a illustrates that the map built by Karto SLAM has many ghosting areas, which might result from the Karto SLAM utilizing window search during iteration. Figure 6b illustrates that Hector SLAM failed to build the map in Scenario 3, and that the yaw from Hector SLAM has obvious errors. Hector SLAM is based on the Gauss–Newton method to match the scan and grid map. When there are not enough features of the external environment, the Gauss–Newton method falls into the local optimal problem.

#### 6.2.2. Positioning Results

**Figure 8:**
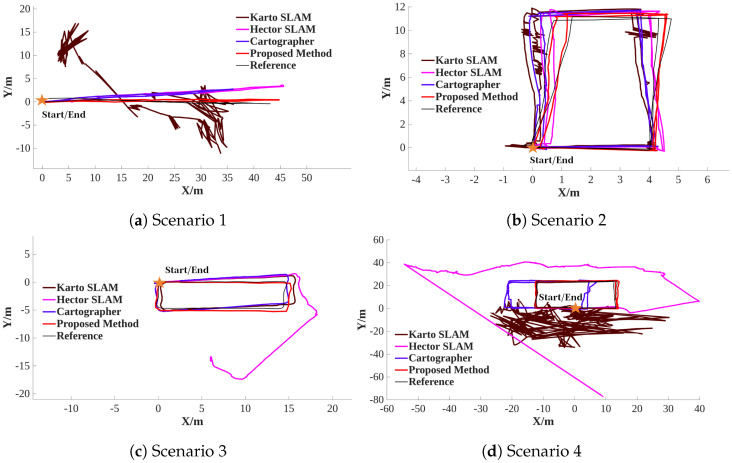
Positioning results.

Figure 8 illustrates the positioning results, which show that the proposed method is closest to the reference trajectory compared with other algorithms. It is observed that the Karto SLAM fails to position in Scenario 1, and there are many burrs in the trajectory of the Karto SLAM in Scenario 2. It is suggested that Karto SLAM has some limitations in practical application. The back-end optimization of Karto SLAM is based on matching submaps, and the optimization method utilizes a multi-scale grid search approach. Compared with the matching laser scan and grid map, the matching submaps method can enrich the features of the scan to some extent, but the multi-scale grid search approach has some limitations; too large an initial search step size will result in the mis-optimization problem. The attitude estimation of Hector SLAM has obvious errors in Scenario 3. The Hector SLAM is based on matching the scan and grid map, and the optimization method is based on the Gauss–Newton method to estimate the robot position (x,y) and the yaw θ. When there are not enough features of the external environment, the Gauss–Newton method easily diverges, especially in the yaw estimation, because yaw estimation is a non-linear problem. In Scenario 4, the proposed method can only provide a reliable positioning service, which suggests that the constraint information from AKF can obviously improve the robustness of the back-end optimization in LiDAR-degenerated environments.

**Figure 9:**
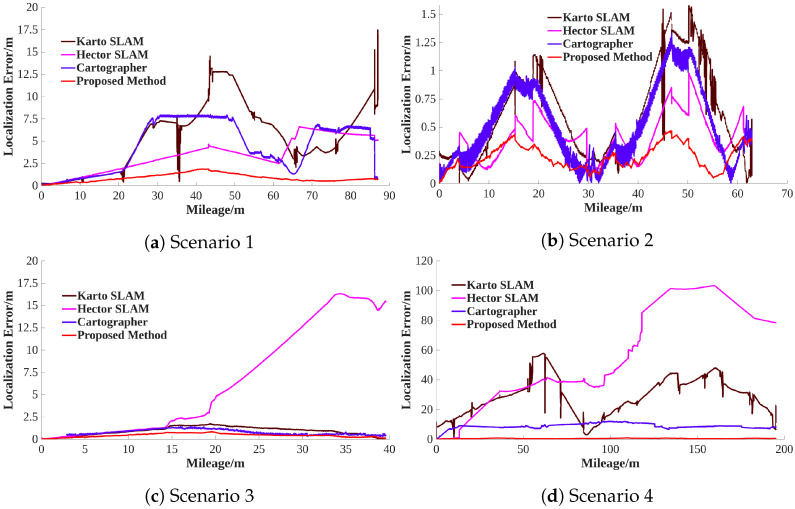
Positioning errors.

Figure 9 demonstrates the positioning errors, and Table 4 illustrates the positioning error static results. It is observed that the proposed method has the highest positioning accuracy in scenarios 1–4. According to Table 4, based on the RMSE, it is found that the positioning accuracy of the proposed method is improved by approximately 61.3–97.9%, 35.7–99.0%, and 43.8–93.0% compared to Karto SLAM, Hector SLAM, and Cartographer, respectively. Especially in Scenario 1, the proposed method improved the positioning accuracy by about 86.6%, 75.4%, and 77.2% compared to Karto SLAM, Hector SLAM, and Cartographer, respectively. In Scenario 4, the positioning accuracy of the proposed method is improved by about 97.9%, 99.0%, and 93.0% compared to Karto SLAM, Hector SLAM, and Cartographer, respectively. It is suggested that the proposed method has obviously improved the robustness of the system in the LiDAR-degenerated environments.

## 7. Discussion

The experiment results indicate that the proposed method has a relatively high robustness in LiDAR-degenerated environments. In the selected environments, the compared algorithms, i.e., Karto SLAM, Hector SLAM, and Cartographer, still have some problems. Scenario 1 is a long corridor, which belongs to a typical LiDAR-degenerated environment. In this scenario, the compared methods all failed to provide a reliable positioning service. The typical problem is that the LiDAR-odometry is less than the actual distance, which is due to the features of the external environment being highly similar, resulting in the error state estimation. Scenario 2 is an indoor hall, which has rich environmental features. Under this case, Hector SLAM and Cartographer have very high performance, but Karto SLAM is not ideal. Karto SLAM optimizes the pose based on the multi-scale search window method. An unreasonable step setting easily results in the local optimization problem. Scenario 3 is a parking lot, which includes many moving objects. Field tests illustrate that Karto SLAM and Cartographer have good performance, but Hector SLAM fails. Hector SLAM is based on the Gauss–Newton method to optimize the pose. Normally, this optimization method has relatively high accuracy, but when the environmental features are not rich enough, or there are too many moving obstacles, it can easily result in mis-optimization, especially in angular estimation. Scenario 4 is a playground, which also belongs to a LiDAR-degenerated environment, but compared with Scenario 1, it has more sparse features. It means that it is more difficult to obtain a reliable positioning estimation according to the LiDAR-odometry. In this section, the parameters of the proposed method are evaluated, including the length of the sliding window for the adaptive factors *L*, and the comparison between the PF-only and AKF+PF.

### 7.1. Influence of the Sliding Window Length *L*

In this section, we analyze the impact of the sliding window length *L* on positioning accuracy, with the root mean square error (RMSE) employed as the static evaluation metric. In this work, the sliding window length *L* is set to 1000 by default, while values of 50, 100, 500, 1000, 1500, and 2000 are also evaluated. The corresponding RMSE results are summarized in Table 5. It can be observed that the sliding window length *L* has a relatively limited influence on positioning accuracy, indicating low error sensitivity to this parameter.

### 7.2. Comparison Between the PF-Only and AKF+PF

In this section, a comparison is made between the PF-only and AKF+PF, with the performance evaluation metric primarily based on the positioning RMSE. The comparison results are tabulated, and the findings are presented in Table 6.

It is found that the AKF+PF has a higher positioning than that of PF-only in the selected scenarios, and the positioning accuracy is improved by about 90.4%, 52.6%, 60.9%, and 97.5%, respectively. It suggests that the fusion of the AKF can improve the positioning accuracy and robustness of the system. The reason for that is that the AKF can provide a reliable estimation of the robot’s pose, and this estimation can avoid mis-optimization during PF optimization, which is obviously after the LiDAR-degenration environments, such as Scenario 1 and Scenario 4.

## 8. Conclusions

In this paper, an innovative 2D-LiDAR SLAM is proposed to solve the issue that the traditional 2D-LiDAR SLAM method has difficulty in providing a reliable positioning service in LiDAR-degenerated scenarios. This method estimates the rough position by fusing the IMU and wheel odometer, and the fusing method is based on the AKF. The rough position will be utilized to predict the particle pose and constrain the back-end optimization. Compared to Karto SLAM, Hector SLAM, and Cartographer, the proposed method improves positioning accuracy by approximately 61.3–97.9%, 35.7–99.0%, and 43.8–93.0%, respectively. It is suggested that, in complex environments, such as LiDAR-degenerated environments like Scenario 1 and Scenario 4, the proposed method is still effective to provide a reliable positioning service. However, there are still some limitations for the proposed method, such as the fact that the wheel odometer can provide accurate linear velocity measurements, but the accuracy of the linear velocity measurement will decrease when the vehicle skids. In addition, the robustness of the proposed method is still low due to the limitation of the single 2D LiDAR’s performance. In the future, a new multi-sensor fusion positioning method will be researched to overcome the problem of vehicle skidding, and the fusion of the multi-2D LiDARs or 3D LiDAR will also be explored to further improve the system’s robustness and build the 3D map.

## Figures and Tables

**Table 1:** Sensor configuration.

Sensors	Configuration
IMU	Frequency: 100 Hz Zero bias stability of gyroscope: 10°/h Zero bias stability of accelerometer: 35 μg Measurement range of gyroscope: 2000°/s Measurement range of acceleration: ±8 g
Wheel Odometer	Frequency: 10 Hz Sensor coupled to wheels: Hall encoder Linear velocity estimation: A skid-steering model based in [19]
2D LiDAR	Frequency: 10 Hz Measurement range: 40 m, when the reflective surface is white Measurement range: 10 m, when the reflective surface is black Angular resolution: 0.391° Distance resolution: 3 cm

**Table 2:** Degree of degeneration.

Scenario	Scenario 1	Scenario 2	Scenario 3	Scenario 4
**Value**	0.91	0	0.58	1

**Table 3:** System comparison framework.

Method	Sensors Used	Front-End Method
Karto SLAM	IMU; Wheel Odometer; LiDAR	AKF
Hector SLAM	IMU; Wheel Odometer; LiDAR	AKF
Cartographer	IMU; Wheel Odometer; LiDAR	Pre-Integration
Proposed Method	IMU; Wheel Odometer; LiDAR	AKF

**Table 4:** Positioning error static results.

	Method	Max	Mean	Median	RMSE
**Scenario 1**	Karto SLAM	17.52 m	6.43 m	6.93 m	7.63 m
	Hector SLAM	6.61 m	3.70 m	4.15 m	4.15 m
	Cartographer	8.00 m	3.37 m	1.98 m	4.47 m
	Proposed Method	**1.87 m**	**0.90 m**	**0.76 m**	**1.02 m**
**Scenario 2**	Karto SLAM	1.60 m	0.64 m	0.58 m	0.76 m
	Hector SLAM	0.98 m	0.37 m	0.40 m	0.42 m
	Cartographer	1.33 m	0.45 m	0.40 m	0.56 m
	Proposed Method	**0.47 m**	**0.25 m**	**0.23 m**	**0.27 m**
**Scenario 3**	Karto SLAM	1.74 m	0.95 m	0.98 m	1.06 m
	Hector SLAM	16.32 m	9.04 m	11.83 m	11.29 m
	Cartographer	1.41 m	0.66 m	0.55 m	0.73 m
	Proposed Method	**0.86 m**	**0.34 m**	**0.33 m**	**0.41 m**
**Scenario 4**	Karto SLAM	57.77 m	19.99 m	17.11 m	25.29 m
	Hector SLAM	103.34 m	41.48 m	38.54 m	55.80 m
	Cartographer	12.12 m	6.47 m	8.03 m	7.72 m
	Proposed Method	**0.96 m**	**0.50 m**	**0.51 m**	**0.54 m**

**Table 5:** Position RMSE under different *L*.

Metric	Values					
*L*	50	100	500	1000	1500	2000
RMSE	0.548 m	0.529 m	0.534 m	0.519 m	0.542 m	0.533 m

**Table 6:** Positioning RMSE comparison results between the PF-only and AKF+PF.

Scenario	Scenario 1	Scenario 2	Scenario 3	Scenario 4
**PF-only**	10.63 m	0.57 m	1.05 m	21.60 m
**AKF+PF**	1.02 m	0.27 m	0.41 m	0.54 m

## Data Availability

The raw data supporting the conclusions of this article will be made available by the authors on request.
